# Glomerulonephritis Histopathological Pattern Change

**DOI:** 10.1186/s12882-020-01836-3

**Published:** 2020-05-18

**Authors:** Anas AlYousef, Ali AlSahow, Bassam AlHelal, Ahmed Alqallaf, Emad Abdallah, Mohammed Abdellatif, Hani Nawar, Riham Elmahalawy

**Affiliations:** 1 Farwaniya Hospital, Department of Internal Medicine, Nephrology Unit, Sabah Al Nasser, Kuwait; 2 Al Jahra Hospital, Department of Internal Medicine, Nephrology Unit, Al Jahra, Kuwait; 3 Al Adan Hospital, Department of Internal Medicine, Nephrology Unit, Hadiya, Kuwait; 4 Mubarak Al-Kabeer Hospital, Department of Internal Medicine, Nephrology Unit, Jabriya, Kuwait

**Keywords:** Diabetic Kidney Disease, Glomerulonephritis, IgA Nephropathy, Kidney Biopsy Nephrotic Syndrome

## Abstract

**Background:**

Glomerulonephritides (GN) are relatively rare kidney diseases with substantial morbidity and mortality. They are often difficult to treat, sometimes with no cure, and can lead to chronic kidney disease (CKD) and end stage kidney disease (ESKD). Kidney biopsy is the diagnostic procedure of choice with variable indications from center to center. It helps in identifying the exact specific diagnosis, assessing the level of disease activity and severity, and hence aids in proper therapy and helps predicting prognosis. There is a global change of pattern of glomerular disease over the last five decades.

**Methods:**

Retrospective analysis of all kidney biopsies (545 cases) that were done in patients over 12 year-old over last six years in four major hospitals in Kuwait. The indications for kidney biopsy were categorized into six clinical syndromes: nephrotic syndrome, sub-nephrotic proteinuria, nephrotic syndrome plus acute kidney injury (AKI), sub-nephrotic proteinuria plus AKI, isolated hematuria, and Unexplained renal impairment. We calculated the incidence of each type of kidney disease and indication of biopsy.

**Results:**

most common indication of kidney biopsy was sub-nephrotic proteinuria associated with AKI in 179 cases (32.8%). Primary Glomerulonephritis was the main diagnosis that was reported in 356 cases (65.3%). Immunoglobulin A Nephropathy (IgAN) was the commonest lesion in primary glomerulonephritis in 85 (23.9%) cases. Secondary Glomerulonephritis was diagnosed in 134 cases (24.6%), 56 (41.8%) of them were reported as lupus nephritis cases. In young adults (below 18 years of age) there were 31 cases reviews, 35.5% were found to have minimal change disease (MCD).

**Conclusion:**

IgAN is the commonest glomerulonephritis in primary nephrotic syndromes in Kuwait over the past six years. Lupus nephritis is the leading secondary glomerulonephritis diagnosis.

## Background

Glomerulonephritides (GN) are relatively rare kidney diseases with substantial morbidity and mortality. They are often difficult to treat, sometimes with no cure, and can lead to chronic kidney disease (CKD) and end stage kidney disease (ESKD). Incidence of ESKD is rising annually worldwide due to a variety of reasons [1, 2]. GN treatment is associated with additional morbidity and cost that is due to expensive immunosuppressive medications, increased risks of infectious, malignancy or other complications. Kidney biopsy is the diagnostic procedure of choice with variable indications from center to center. It is useful in identifying the exact specific diagnosis, assessing the level of disease activity and severity, and hence aids in proper therapy and helps predicting prognosis. Common clinical scenarios where biopsy is needed are nephrotic syndrome (NS), prolonged acute kidney injury (AKI), rapidly progressive glomerulonephritis (RPGN), systemic diseases with renal dysfunction, sub-nephrotic proteinuria, isolated microscopic hematuria, unexplained renal impairment, renal transplant dysfunction, and some familial renal diseases. Kidney biopsy data can help identify prevalence of certain renal diagnoses. There is a global change of pattern of glomerular disease over the last five decades. Here we present our data of six years of native kidney biopsies in four centers in Kuwait comprising about 80% of all cases in the country.

## Methods

All reports of native kidney biopsies performed in four public hospitals from January 2013 to December 2018 were retrospectively analyzed. Minimal age of patients to be involved in the study was 12 years old (Pediatric age in Kuwait is up to 12 year-old). We recorded the following data for each patient: name, age, sex, indication of kidney biopsy, histopathological diagnosis and laboratory investigations such as serum creatinine, 24-hour urinary protein, urine microscopy, virology; hepatitis B surface antigen (HBsAg), hepatitis C antibody (anti-HCV), Human Immunodeficiency Virus (HIV) and serology; anti-double stranded DNA antibody, antinuclear antibody (ANA), complements levels 3&4 (C3, C4). All kidney biopsy specimens obtained were prepared as per the standard protocol and examined by the same renal pathologists located in one hospital. Analysis included light microscopy (LM) and immunofluorescence (IF). However, electron microscopy (EM) was not systematically performed, as this facility was not widely available during that period. Tissue processing was performed from formalin-fixed and paraffin-embedded (FFPE) tissue. Tissue sections were cut at 4μm thickness. Staining included a hematoxylin and eosin (HE), a periodic acid-Schiff (PAS), a silver stain (Jones’ Methenamine) and a Masson's trichrome stain. Special stains were used when warranted. Immunoglobulins and complement analyses were done using an immunohistochemical antibody panel to immunoglobulins G, M, A (IgG, IgM, IgA), C3, complement 1 q (C1q), and, if needed, to kappa and lambda light chains. The indications for kidney biopsy were categorized into six clinical syndromes: nephrotic syndrome, sub-nephrotic proteinuria, nephrotic syndrome plus AKI, sub-nephrotic proteinuria plus AKI, isolated hematuria, and Unexplained renal impairment. In CKD patients with unexplained deterioration in kidney function, kidney biopsy was performed if kidney sizes were within normal limits by ultrasound with intact corticomedullary differentiation. Automated biopsy guns were used for all biopsies.

Histological categories were classified as follows: 1) glomerular disease. 2) non-glomerular diseases like tubulointerstitial diseases. 3) normal kidneys. 4) insufficient material for analysis. Those patients diagnosed with glomerular diseases were further divided into having primary or secondary glomerular diseases. Extensive glomerulosclerosis, severe tubular loss and/or atrophy, some degree of cystic changes and thickened renal blood vessels were considered as suggestive of ESKD. We calculated the incidence of each type of kidney disease and indication of biopsy. Comparison was made between data from each year from 2013 to 2018. The data generated and analyzed were also compared with studies published from Kuwait and different regions of the world.

Simple descriptive statistics such as median and mean were used for variables such as age, clinical and laboratory features. Percentage was used for categorical data.

### Definitions

Nephrotic syndrome: proteinuria of more than 3 g per day associated with hypoalbuminemia. Sub-nephrotic proteinuria: proteinuria of less than 2 g per day. Acute kidney injury was defined as an increase in serum creatinine of ≥ 26.5 μmol/L within 48 hours, or increase in serum creatinine ≥ 1.5 times baseline which is known or presumed to have occurred within the prior 7 days, or urine volume <0.5 ml/kg/h for 6 hours [3]. Unexplained renal impairment was diagnosed in patients presenting with persistently elevated serum creatinine with no obvious cause that is associated with or without hematuria or proteinuria, and no history of diabetes or hypertension.

## Results

Total of 545 cases were available for review. There were only 120 patients with history of diabetes (21.9%), and 178 cases were known hypertensive (32.5%). Male patients were predominant at 335 cases (61.2%). Mean age of patients at time of biopsy was 39.8 years. Youngest patient was at 12 years old and the oldest patient was a 90-year-old male patient. There were only three cases with HCV positive antibody with a diagnosis of: diabetic nephropathy, membranous nephropathy and extensive glomerulosclerosis. Also we had three cases that had HBsAg positivity their biopsy results showed: minimal change disease, membranous nephropathy, and diabetic nephropathy. There were no HIV positive patients in the biopsy cohort.

**Fig. 1:**
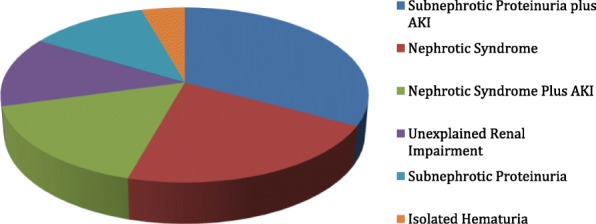
Indications of Kidney Biopsy

The most common indication of kidney biopsy was sub-nephrotic proteinuria associated with AKI in 179 cases (32.8%). Nephrotic syndrome followed with 117 cases (21.5%). Nephrotic syndrome associated with AKI came third with 89 cases (16.3%). Least indication for kidney biopsy was isolated hematuria with only 24 cases (8%). There were 71 (13%) cases that got biopsied due to unexplained renal impairment (Table 1 & Fig 1).

**Table 1:** Indications of Kidney Biopsy (Clinical Presentation)

Clinical Syndrome	2013	2014	2015	2016	2017	2018	Total N	Percent
Sub-nephrotic Proteinuria plus AKI	11	22	52	32	23	39	179	32.8 %
Nephrotic Syndrome	8	20	18	12	29	30	117	21.5 %
Nephrotic Syndrome plus AKI	8	11	12	19	22	17	89	16.3 %
Unexplained Renal Impairment	10	7	11	20	9	14	71	13 %
Sub-nephrotic Proteinuria	7	8	23	3	10	14	65	11.9 %
Isolated Hematuria	2	2	6	5	1	8	24	4.4 %

Primary Glomerulonephritis was the main diagnosis that was reported in 356 cases (65.3%). IgAN was the commonest lesion in primary GN in 85 (23.9%) cases. Membranous nephropathy (MN) and focal segmental glomerulosclerosis (FSGS) came second and third in 79 (22.2%) and 78 (21.9%) cases, respectively (Table 2). Secondary Glomerulonephritis was diagnosed in 134 cases (24.6%), 56 (41.8%) of them were reported as lupus nephritis cases, and 73% of these lupus nephritis cases were females. Diabetic kidney disease followed with 27 (20.2%) cases (Table 3). Tubulointerstitial nephritis (TIN) cases were 45 where 66.7% of them were acute interstitial nephritis (AIN) (Table 4).

**Table 2:** Primary Glomerulonephritis

Glomerulopathy Type	Number of cases	Percent
IgAN	85	23.9 %
Membranous Nephropathy	79	22.2 %
FSGS	78	21.9 %
MCD	50	14.0 %
MPGN	11	3.1 %
Crescentic GN	21	5.9 %
Extensive Glomerulosclerosis	24	6.7 %
Other primary GN	8	2.2 %
Total	356	100.0

**Table 3:** Secondary Glomerulonephritis

Glomerulopathy Type	Number of cases	Percent
Lupus Nephritis	56	41.8
Diabetic Kidney Disease	27	20.2
ANCA Vasculitis	13	9.7
TMA	12	8.9
Amyloidosis	6	4.5
Post infectious GN	6	4.5
Hypertensive Nephropathy	9	6.7
Light chain cast nephropathy	5	3.7
Total	134	100.0

**Table 4:** Tubulointerstitial Disease

Tubulointerstitial	Number of cases	Percent
Acute interstitial nephritis	30	66.7
Chronic interstitial nephritis	10	22.2
Other tubulointerstitial disease	5	11.1
Total	45	100.0

Further analysis was done with regards to findings under each category. Sub-nephrotic proteinuria associated with AKI cases were 179, IgAN were present in 42 (23.4%) cases, followed by FSGS with 37 (20.7%) cases. Only 15 (8.4%) cases in this category were due to lupus nephritis. Top three diagnoses in nephrotic syndrome category were MN (30.8%), followed by minimal change disease (MCD) (29.1%), FSGS (12.8%), and lupus nephritis (11.1%). Peak age at presentation was 66 years for MN patients, 55 years for FSGS, and 55 years for MCD. There were 34% of cases of MN with eGFR below 60 ml/min/m^2^ at time of biopsy. Nephrotic syndrome associated with AKI was mainly reported as MN in 24 (27%) cases then FSGS (12.4%) and IgAN (11.2%). In 71 cases that got biopsied due to unexplained renal impairment, majority was AIN in 18 (25.4%) cases. Lupus nephritis was diagnosed in 13 (20%) of 65 cases presenting with sub-nephrotic proteinuria; Followed by FSGS and MN each in 11 cases (17%) then IgAN with 9 cases (13.8%). IgAN dominated isolated hematuria cases in 10 out of 24 cases (41.7%) (Table 5).

**Table 5.** Clinical Presentation and Correlative Histopathology

Diagnosis	Sub-nephrotic Proteinuria plus AKI	Nephrotic syndrome	Nephrotic Syndrome plus AKI	Sub-nephrotic Proteinuria	Isolated Hematuria	Unexplained renal impairment
Lupus Nephritis	16	14	7	13	2	4
FSGS	37	15	11	11	0	4
MN	6	36	24	11	1	1
MCD	2	34	6	8	0	0
IgAN	42	8	10	9	11	5
MPGN	3	1	4	1	1	1
ANCA Vasculitis	1	0	0	3	1	8
Crescentic GN	11	2	7	0	1	0
DKD^1^	16	1	7	2	0	6
TMA^2^	7	0	0	1	2	2
AIN	11	0	0	1	0	18
CIN^3^	5	0	1	1	0	3
Extensive glomerulosclerosis	11	1	2	0	0	5
HTN Nephropathy	3	0	0	0	1	5
Alport Syndrome	1	1	0	0	0	0
Inadequate Biopsy	0	0	0	1	0	5
C3 Glomerulopathy	0	0	0	0	0	1
IgG4	0	0	0	0	0	1
Thin Basement Membrane	0	0	0	1	1	0
Granulomatous Nephritis	0	0	0	0	0	1
Renal amyloidosis	1	2	3	0	0	0
Post infectious GN	4	0	0	0	2	0
Pigment nephropathy	1	0	0	0	0	0
Light chain cast nephropathy	1	0	4	0	0	0
Normal kidney biopsy	0	3	0	0	1	0
Lymphomatous Infiltrate (CLL)	0	0	1	0	0	0
Tubular adenoma	0	0	1	0	0	0
Fibrillary GN	0	0	1	0	0	0
Total	179	117	89	65	24	71

(1) *DKD* Diabetic Kidney Disease. (2) *TMA* Thrombotic Microangiopathy. (3) *CIN* Chronic Interstitial Nephritis.

Total of 120 biopsies were done in diabetic patients for a variety of indications. The commonest indication for kidney biopsy in diabetic patients was sub-nephrotic proteinuria associated with AKI in 45.8% of the cases, followed by unexplained deterioration in kidney function in 27.5% of the cases. The commonest diagnosis was diabetic kidney disease in 27 (22.5%) cases followed by FSGS 23 (19.2%) cases, and later came equally IgAN and extensive glomerular sclerosis in 13 (10.8%) cases each. Lupus nephritis and AIN were present in 7.5% and 5% of cases, respectively (Table 6).

**Table 6:** Histopathological Patterns in Diabetic Patients

Diagnosis	Number of cases	Percent
Diabetic Kidney Disease	27	22.5 %
FSGS	23	19.2 %
IgA Nephropathy	13	10.8 %
Extensive glomerulosclerosis	13	10.8 %
Lupus nephritis	9	7.5 %
Membranous Nephropathy	7	5.8 %
MCD	3	2.5 %
MPGN	2	1.7 %
Focal necrotizing GN	4	3.3 %
Crescentic GN	3	2.5 %
AIN	6	5 %
HTN Nephropathy	3	2.5 %
Thrombotic Microangiopathy	1	0.8 %
C3 Glomerulopathy	1	0.8 %
Chronic Interstitial Nephritis	1	0.8 %
Renal Amyloidosis	1	0.8 %
Post infectious glomerulonephritis	1	0.8 %
Inadequate Biopsy	2	1.7 %
Total	120	100 %

Cases done in young adults (12 to 18 years of age) were 31 in total. The main indications for biopsy were sub-nephrotic proteinuria, nephrotic syndrome, and sub-nephrotic proteinuria associated with AKI. The commonest finding in this population was MCD in 11 (35.5%) cases (Table 7)

**Table 7:** Clinical Presentations and Histopathological Results in patients less than 18 years

**(1) Indications of Kidney Biopsy**		
**Indication**	**Number of Cases**	**Percent**
Sub-nephrotic Proteinuria	11	35.5 %
Nephrotic Syndrome	9	29 %
Sub-nephrotic Proteinuria plus AKI	7	22.6
Nephrotic Syndrome plus AKI	2	6.5 %
Unexplained deterioration in kidney function	2	6.5 %
Total Number	31	100%
**(2) Results of Kidney Biopsy**		
**Diagnosis**	**Number of Cases**	**Percent**
MCD	11	35.5 %
MPGN	3	9.7 %
IgA Nephropathy	3	9.7 %
Lupus nephritis	3	9.7 %
Membranous Nephropathy	2	6.5 %
FSGS	2	6.5 %
AIN	2	6.5 %
Crescentic GN	1	3.2 %
Post infectious GN	1	3.2 %
Chronic Interstitial Nephritis	1	3.2 %
Focal necrotizing GN	1	3.2 %
Alport Syndrome	1	3.2 %
Total Number	31	100%

## Discussion

**Fig. 2.**
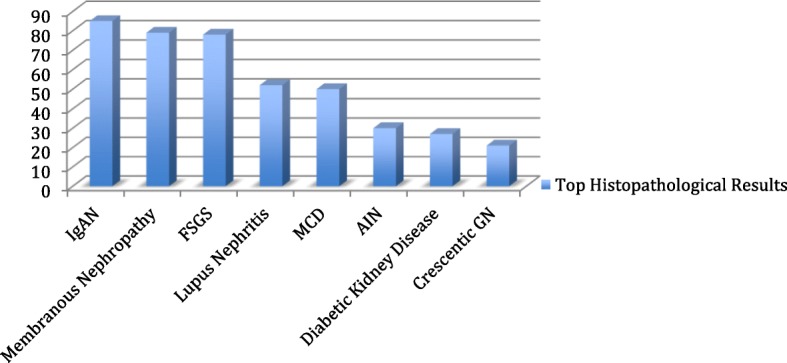
Top Histopathological Results

There has been a global change in histological pattern of GN over past 5 decades. There was a point in time were MN was the commonest primary nephrotic syndrome [4]. This was changed, as FSGS took over the lead since mid 1990s [4]. At present it is well known that IgAN is the commonest GN diagnosed on kidney biopsies, regardless of the presentation, level of kidney function, or indication of kidney biopsy [5, 6]. A recent analysis that was done in India between 2002 and 2015 in a single center found that IgAN was present in 21.6% of kidney biopsy specimens [7]. Our study goes in the same line with the current trend; IgAN is the commonest GN in Kuwait clinically presented as sub-nephrotic proteinuria associated with AKI in majority of cases (Fig 2).

There were two single centered studies carried out in Kuwait looking at GN prevalence. First one was done between 1995-2001 in one center in Kuwait. They found FSGS to be the most common histological lesion accounting for 18.0% of the total biopsies. Minimal change disease was the second primary GN (13.0%), followed by immunoglobulin A deposition disease (7.9%) and membranous glomerulonephritis (5%) [8]. These results were compatible with the international findings at that time. Data from 1970s have indicated that MN was the main primary GN in idiopathic NS but in mid 1990s FSGS took the lead as the most common GN, and this period saw also a rise in cases of IgAN [4]. Another single center study during the period from January 1986-December 2002 showed FSGS to be the main GN followed by IgAN [9]. FSGS remained the leading GN up until late 2000s. In a neighboring country, Saudi Arabia, single center analysis was done between Jan 2005 to December 2009 which showed the most common histological lesion was FSGS (27.6%), followed by minimal change disease (17.7%) and membrano-proliferative glomerulonephritis (MPGN) (13.0%) [10]. Regionally, a cohort in Egypt was analyzed for the period from July 2003 to July 2008. Focal segmental glomerulonephritis was the most frequent cause of primary GN (21.21%), followed by mesangial proliferative GN (18.93%), diffuse proliferative GN (13.96%), focal proliferative GN (12.77%) and membranous GN (10.93%) [11].

The other single center study from Kuwait, about a decade after the first one, looked at kidney biopsies between 2009-2014 showed membranous GN to be the most common lesion (12.1%), followed by IgAN (11.7%), minimal change disease (9.8%), focal and segmental glomerulosclerosis (9.3%) [12]. Percentages were close by where one can notice a rising trend in IgAN.

Kuwait has diversity of populations living together where about 70% of the residents are expatriates from close by countries like the Middle East and South East Asia. However, this cohort represents equal number of nationals and expatriates. About half of the total number of IgAN cases, as well as other histological diagnoses in this cohort where in Kuwaiti nationals. The difference in the histological patterns of GN found in this cohort in comparison to the other two mentioned earlier can not be attributed completely to ethnic background [8, 12].

Lupus nephritis remains the commonest secondary GN and this finding is universal throughout the world [13–15]. However, the number of males with lupus nephritis in our cohort is large (27%). Males represent 4-22% of SLE patients in reported cases [16], with higher percentage for males in lupus nephritis [17]. Acute interstitial nephritis is not an uncommon pathology. There were 30 cases of AIN in the whole cohort (5.5%) and six cases (5%) among diabetic patients, more than half of them presented with unexplained renal failure. One needs to be vigilant about new medications like antibiotics, proton pump inhibitors and over the counter painkillers as these are the common causes of AIN. It is becoming increasingly more prevalent in recent analyses that might be due to more aggressive diagnostic measures being undertaken [14].

**Fig. 3.**
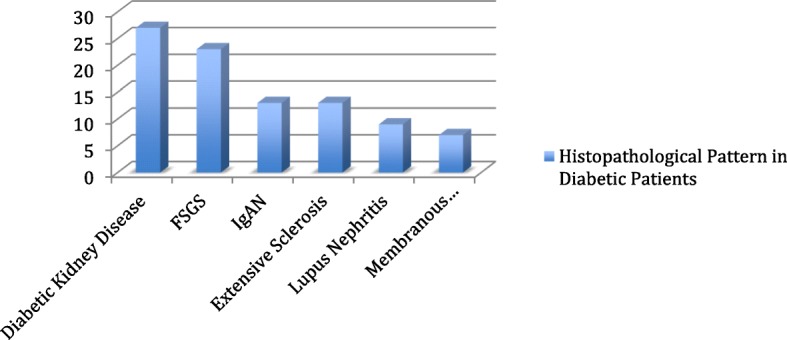
Histopathological Pattern in Diabetic Patients

The prevalence of diabetes in Kuwait is high, ranked as one of the top ten countries worldwide in prevalence of diabetes [18]. There were 120 (22%) cases done on patients with history of diabetes. About a third of all cases were found to be having diabetes related disease, like diabetic kidney disease (22.5%). Two thirds of the kidney biopsies done in diabetic patients showed non-diabetic related diseases like FSGS, IgAN, lupus nephritis, and others (Table 3 & Fig 3). This is an important finding indicating a high prevalence of other than diabetic lesions in kidney biopsies in diabetic patients where other diagnoses could be entertained and therapy is instituted. Similar findings were published in a study from New York where more than 60% of biopsies done in diabetic patients showed non diabetic related diseases [19].

This study helps in acknowledging the global change in histological pattern in GN. Kidney biopsy is the gold standard diagnostic test and is a powerful tool that should be widely used in cases where renal diagnosis is uncertain, and renal prognosis is warranted before recommending an expensive, possibly toxic therapy. GN registry would be of utmost importance in the future for both diagnostic and therapeutic implications. By diagnosing and maybe understanding pathophysiology of some GN cases one can design an earlier diagnostic assay or measure that would help carrying out quick therapeutic measures to ameliorate associated morbidity and mortality in such cases.

Major strength of our study is the multi-center, large sample size, and the relatively long duration that allow for multiple different presentations in glomerular diseases as we saw in other earlier studies that showed changing leading causes of primary nephrotic syndrome worldwide. Also this study is the largest in the region, which could help in future research on development of further diagnostic and therapeutic advancements. Limitations of the study are: 1) The retrospective nature of the study. 2) Absence of electron microscopy for most of the duration of the study period. If it was available it might have added further data on some diagnoses but would not necessarily have affected the final diagnosis in the majority of cases [20].

## Conclusion

Commonest indication for kidney biopsy in Kuwait is sub-nephrotic proteinuria associated with AKI. IgAN is the commonest GN in primary nephrotic syndromes in Kuwait over the past six years. Lupus nephritis remains the leading secondary GN diagnosis. AIN is not an uncommon diagnosis and should be highly suspected in cases of unexplained deterioration of kidney function. In diabetic patients, kidney biopsy in the right settings can detect significant different etiologies other than diabetic kidney disease.

## Data Availability

The datasets generated during and/or analyzed during the current study are available from the corresponding author on reasonable request.

## Abbreviations

GN: Glomerulonephritides
CKD: Chronic kidney disease
ESKD: End stage kidney disease
NS: Nephrotic syndrome
AKI: Acute kidney injury
RPGN: Rapidly progressive glomerulonephritis
HBsAg: Hepatitis B surface antigen
anti-HCV: Hepatitis C antibody
HIV: Human immunodeficiency virus
ANA: Antinuclear antibody
C3: Complement levels 3
C4: Complement levels 4
LM: Light microscopy
IF: Immunofluorescence
EM: Electron microscopy
FFPE: Formalin-fixed and paraffin-embedded
HE: Hematoxylin and eosin
PAS: Periodic acid-Schiff
IgG: Immunoglobulin G
IgM: Immunoglobulin M
IgA: Immunoglobulin A
C1q: Complement 1 q
MN: Membranous nephropathy
FSGS: Focal segmental glomerulosclerosis
TIN: Tubulointerstitial nephritis
AIN: Acute interstitial nephritis
MPGN: Membrano-proliferative glomerulonephritis
IgAN: Immunoglobulin a nephropathy
